# A Novel Phosphoglucomutase-3 Gene Variant Causing Milder Phenotype in Two Families

**DOI:** 10.1007/s10875-026-02012-2

**Published:** 2026-03-29

**Authors:** Betul Gemici Karaaslan, Mehdi Benamar, Selami Ulas, Sezin Aydemir, Gozde Yesil, Alper Gezdirici, Aysun Ayaz Sari, Serdar Nepesov, Haluk Cokugras, Yildiz Camcioglu, Akif Ayaz, Cigdem Aydogmus, Louis-Marie Charbonnier, Talal Chatila, Ayca Kiykim

**Affiliations:** 1 Department of Pediatric Immunology and Allergy, School of Medicine, Istanbul University-Cerrahpaşa, Kocamustafapasa, Fatih Istanbul, 34098 Türkiye; 2 Division of Immunology, Boston Children’s Hospital, Boston, MA USA; 3 Department of Pediatrics, Harvard Medical School, Boston, MA USA; 4 Institute of Regenerative Medicine and Biotherapy, INSERM, University of Montpellier, Montpellier, France; 5 Department of Pediatric Immunology and Allergy, University of Health Sciences, Basaksehir Cam and Sakura City Hospital, Istanbul, Türkiye; 6 Department of Medical Genetics, Istanbul University Faculty of Medicine, Istanbul, Türkiye; 7 Department of Medical Genetics, University of Health Sciences, Basaksehir Cam and Sakura City Hospital, Istanbul, Türkiye; 8 Department of Pediatric Neurology, School of Medicine, Istanbul University-Cerrahpaşa, Istanbul, Türkiye; 9 Genetic Diseases Assessment Center, Memorial Health Group, Şişli Hospital, Istanbul, Türkiye

**Keywords:** Phosphoglucomutase-3, Hyper IgE syndrome, Phosphoflow assay

## Abstract

**Background:**

Phosphoglucomutase 3 deficiency (PGM3 deficiency) is a rare congenital disorder of glycosylation classically associated with severe immunodeficiency, skeletal abnormalities, and neurodevelopmental impairment. However, emerging evidence suggests that PGM3 deficiency may also present with attenuated or milder clinical phenotypes. In this study, we describe patients with a novel PGM3 variant exhibiting a less severe immunological and clinical presentation, thereby expanding the known phenotypic spectrum of PGM3 deficiency.

**Methods:**

Demographic-data and Wechsler Intelligence Scale for Children profiles were evaluated alongside laboratory data, including complete blood cell counts, lymphocyte subsets, serum immunoglobulin levels, vaccine antibody titers, and lymphocyte cytokine profiles. Whole exome sequencing was conducted, and phospho-flow assays were utilized for the analysis of p-STATs.

**Results:**

Five patients with a mild form of PGM3 deficiency were described, exhibiting a Hyper-IgE Syndrome phenotype without severe skeletal dysplasia or dysmorphism, with the exception of one patient displaying very mild skeletal dysplasia and three patients exhibiting mild to moderate intellectual disability. All patients demonstrated an increase in Natural Killer T cells and a decrease in B cells, alongside an increase in activated CD4 + T cells (CD45RO+), a decrease in naïve CD4 + and CD8 + T cells (CD45RA+ CCR7+), and an increase in TEMRA CD8 + T cells (CD45RA+CCR7–). Functional analysis of all patients revealed impaired PGM3 function, as evidenced by decreased surface expression of gp130 and p-STAT3.

**Conclusion:**

Defective glycosylation in PGM3 deficiency leads to reduced gp130 expression and attenuated gp130-dependent STAT3 phosphorylation. The resulting cellular features partially overlap with those observed in STAT3 loss-of-function and gp130 deficiency, supporting a shared signaling mechanism while remaining clinically distinct.

**Supplementary Information:**

The online version contains supplementary material available at 10.1007/s10875-026-02012-2.

## Introduction

Phosphoglucomutase 3 (PGM3) is an important enzyme in both N- and O-linked glycosylation pathways. PGM3 converts N-acetylglucosamine-6 to N-acetylglucosamine-1, a critical sugar nucleotide. This converting step is necessary to produce uridine diphosphate N-acetylglucosamine (UDP-GlcNAc), an essential molecule of glycolipids, proteoglycans, and glycosaminoglycans [1].

Mutations of genes involved in glycan metabolism (synthesis and branching) cause a heterogeneous group of rare inherited diseases named congenital disorders of glycosylation (CDG) [2]. CDG shows broad spectrum typical features such as neurologic deficits, hematologic abnormalities, dysmorphism, malformations, and abnormal immune functions due to its essential roles in normal cellular functions [1, 3]. These patients have different phenotypes and disease severity independently of subtype, even among patients from the same family. Although the hypothesis is unproven that the differences have been associated with polymorphism in other genes in glycosylation, it may have a role in phenotypical differences of the patients [4]. Congenital disorders of glycosylation (CDGs) have been described to lead to primary immune deficiencies (PID). Since accurate glycosylation of most immune receptors, immunoglobulins, proteins of the complement and cytokines is essential for the integrity of the immune functions [5].

In 2014, PGM3 deficiency was described by three independent groups [3, 5, 6]. Since the first description, 46 patients from 22 kindreds have been reported [3, 5–16]. The patients with PGM3 deficiency presented basically three different phenotypes: primary atopic disorders including, Hyper IgE syndrome (HIES) [17] and combined immunodeficiency characterized by elevated serum IgE levels, eczema, recurrent skin and pulmonary infections with or without syndromic features such as variable skeletal and connective tissue abnormalities, has been reported in patients with PGM3 deficiency, often accompanied by additional features such as neurological impairment, dysmorphic features, skeletal dysplasia, or neutropenia; severe combined immunodeficiency (SCID) with profound T cell lymphopenia, prominent skeletal features, and neurologic impairments and combined immunodeficiency (CID) [3, 5–16, 18]. However, the relationship of these broad phenotypic presentations with the degree of impairment in PGM3 enzymatic function and the correlation between *PGM3* genotype and clinical phenotypes remain to be established [8, 16, 19].

Herein, we investigated five patients from two unrelated families with PGM3 deficiency carrying the same homozygous PGM3 variant. All patients experienced infections of variable severity, lymphopenia, and heterogeneous serum IgE levels, but none exhibited dysmorphic features or severe neurodevelopmental impairment. Three patients had mild learning difficulties, and one patient showed skeletal involvement limited to degenerative changes of the metatarsal–metacarpal joints. Importantly, none of the patients fulfilled criteria for severe combined immunodeficiency or required hematopoietic stem cell transplantation. Taken together, these findings indicate an attenuated clinical presentation, characterized by reduced skeletal and neurodevelopmental involvement compared with previously reported severe PGM3 phenotypes associated with other variants. Accordingly, this study aimed to evaluate the clinical and immunological attributes that are associated with this mild *PGM*3 mutation in an attempt to better understand phenotypic/genotypic relationships associated with PGM3 deficiency.

## Materials and Methods

### Study Participants

Medical data of all patients were obtained by direct interview with parents and investigating in medical records after receiving written informed consent from parents or guardians. Clinical diagnosis of combined immunodeficiency (CID) has been established according to the European Society for Immunodeficiencies criteria [20]. Peripheral blood samples were obtained in sodium heparin or ethylenediaminetetraacetic acid (EDTA) tubes at study enrollment. Peripheral blood mononuclear cells (PBMCs) were isolated from whole blood via density gradient using Ficoll (GE Healthcare). PBMCs were then stored frozen in Fetal Calf Serum (FCS) (Sigma Aldrich) and 15% Dimethyl sulfoxide (DMSO) (Sigma Aldrich). The cells were later thawed for analysis by flow cytometry.

### Clinical Data and Flow Cytometric Analysis

Demographic data included age, sex, first presentation, age at disease onset and diagnosis, outcome, and Wechsler Intelligence Scale for Children (WISC-R) profile evaluation. The laboratory data included complete cell blood counts, lymphocyte subsets (by flow cytometry), serum immunoglobulins (assessed using nephelometry), antibody titers to vaccinations, and lymphocyte-cytokine profile assays. Peripheral lymphocyte subset analyses were performed by flow cytometry [21].

To analyze different lymphocyte subsets, the following monoclonal antibodies (mAbs) were used: CD3—APC/Cyanine7 (UCHT1, Catalogue no: 300426, Biolegend), CD4—Brilliant Violet 785 (OKT4, Catalogue no: 317442, Biolegend), CD8—Brilliant Violet 605 (SK1, Catalogue no: 337212, Biolegend), CCR6—Brilliant Violet 711 (G034E3, Catalogue no: 353436, Biolegend), Foxp3- eFluor 450 (PCH101, Catalogue no: 48–4776-42, Thermofisher), CD45RA— Brilliant Violet 605 (HI100, Catalogue no: 304134, Biolegend), CD45RO— Brilliant Violet 711 (UCHL1, Catalogue no: 304236, Biolegend), FITC CRTH2 (BM16, Biolegend), APC-Gata3 (16E10A23, Biolegend), BV605 CCR4 (L291H4, Biolegend), PE RORγT (Q31-378, BD Biosciences), CD56 (QA17A16, Biolegend), CD19 (HIB19, Biolegend), CD25 (BC96, Biolegend), CD127 (A019D5, Biolegend), CCR7 (G043H7, Biolegend), gp130 (2E1B02, Biolegend), CD161 (HP-3G10, Biolegend). For intracellular IL-4, IL-17 A, IL-2 and IFN-γ detection, cell suspensions (1 × 10^6^ cells) were incubated with protein transport inhibitor containing monensin (BD Bioscience, USA), PMA (50 ng/ml) and ionomycin (1 µg/ml) for 6 hours. Dead cells were distinguished from live cells using Fixable Viability Dye eFluor 506 (1:1000 dilution, Catalogue no: 65–0866-14, Thermofisher). Surface and intracellular markers were stained as previously described. Data was collected from stained cells by using Fortessa cytometer (BD Biosciences) and was analyzed by using FlowJo software (v10.10, Tree Star Inc.).

### Analysis of p-STATs by Phosphoflow

Total PBMCs (1 × 106) from either healthy controls or patients were stimulated at 37 °C in non-supplemented RPMI 1640 with IL-21 (20 ng/ml, *Peprotech*, Cat#:200-21-2UG) in 96 well plate. The reaction was stopped and cells were permeabilized using a Foxp3/transcription factor staining buffer (eBiosciences) and Perm buffer III (BD Biosciences). Cells were stained using P-STAT3 (A15137E, Biolegend, APC-Cy7 CD3 (OKT3, Biolegend), BV785 CD4 (OKT4, Biolegend). Samples were acquired on a Fortessa cytometer (BD) and data were analyzed using the FlowJo software.

### Molecular and Functional Assays

Genomic DNA was extracted from peripheral blood using MagnaPure X blood extraction kit according to standard protocols (Roche Diagnostics). Whole exome sequencing was performed for the proband in family A using Nextera Rapid Capture Enrichment (Illumina) kit. For each subject, 50 ng of genomic DNA was used to prepare a captured library that was then sequenced on a NextSeq500 platform (Illumina), generating 150 bp paired-end reads. Raw data of approximately 10 GB per exome were mapped to a human reference genome sequence (GRCh37/hg19) using the Burrows–Wheeler Alignment (BWA) tool [22]. Variant calling was performed using the Genome Analysis Toolkit (GATK) [23]. All variants were further annotated with ANNOVAR software [24]. Variant frequency was evaluated with public databases such as Exome Aggregation Consortium (ExAC), 1000 Genomes Project, and GnomAD [25]. For functional prediction of single-nucleotide variants (SNVs), SIFT [26], PolyPhen-2 [27] and MutationTaster [28] databases were used. The Sanger sequencing technique was used to confirm the candidate variant.

Candidate variants were prioritized using a multi-step filtering strategy. Initial selection was restricted to high-quality variants (read depth > 10×, Genotype Quality > 45) with a minor allele frequency (MAF) < 1% in the gnomAD database. Analysis was further limited to high- and moderate-impact variants (including frameshift, nonsense, missense, and splice-site alterations), while excluding those classified as ‘benign’ or ‘likely benign’ in ClinVar. Subsequent filtering based on the HPO term ‘Immunodeficiency’ (HP:0002721) yielded two candidates. The primary variant identified was a homozygous missense variant in PGM3 (NM_001199917.1:c.799G > T; p.Asp267Tyr). According to ACMG criteria, this variant was classified as a Variant of Uncertain Significance (VUS) based on PM2, PM5, and PP3 evidence. The variant was absent from population databases, including gnomAD and the Turkish Variome database (PM2). A different missense change affecting the same amino acid residue has previously been reported as pathogenic (ClinVar Variation ID: 224830), supporting PM5. Multiple in silico prediction tools (SIFT, PolyPhen-2, PROVEAN, MetaRNN, and CADD) consistently indicated a deleterious effect (PP3). Additionally, the affected residue is highly conserved across species (PhyloP score: 6; PhastCons score: 1).

Apart from the primary variant identified in the PGM3 gene, the filtering process identified a heterozygous c.359 C > T (p.P120L) variant in the TYK2 (NM_003331.4) gene. While TYK2 mutations are associated with autosomal recessive Immunodeficiency 35, this specific variant is currently classified as a Variant of Uncertain Significance (VUS) in ClinVar. Given its heterozygous state and VUS classification, it was not considered to contribute to the patient’s phenotype (Table S1).

### Statistical Analysis

Statistical analyses were conducted using GraphPad Prism software (GraphPad Software, San Diego, CA, USA). Flow cytometry data are shown as individual data points, with summary results expressed as mean ± standard error of the mean (SEM). Comparisons between patients with PGM3 deficiency and healthy controls were performed using appropriate parametric tests when applicable. For non-parametric group comparisons, a two-tailed Mann–Whitney U test was applied. A P value < 0.05 was considered statistically significant. Significance was indicated as follows: *P < 0.05, **P < 0.01, and ***P < 0.001. The number of samples analyzed in each experiment is specified in the corresponding figure legends.

## Results

### Case Reports

**Fig. 1:**
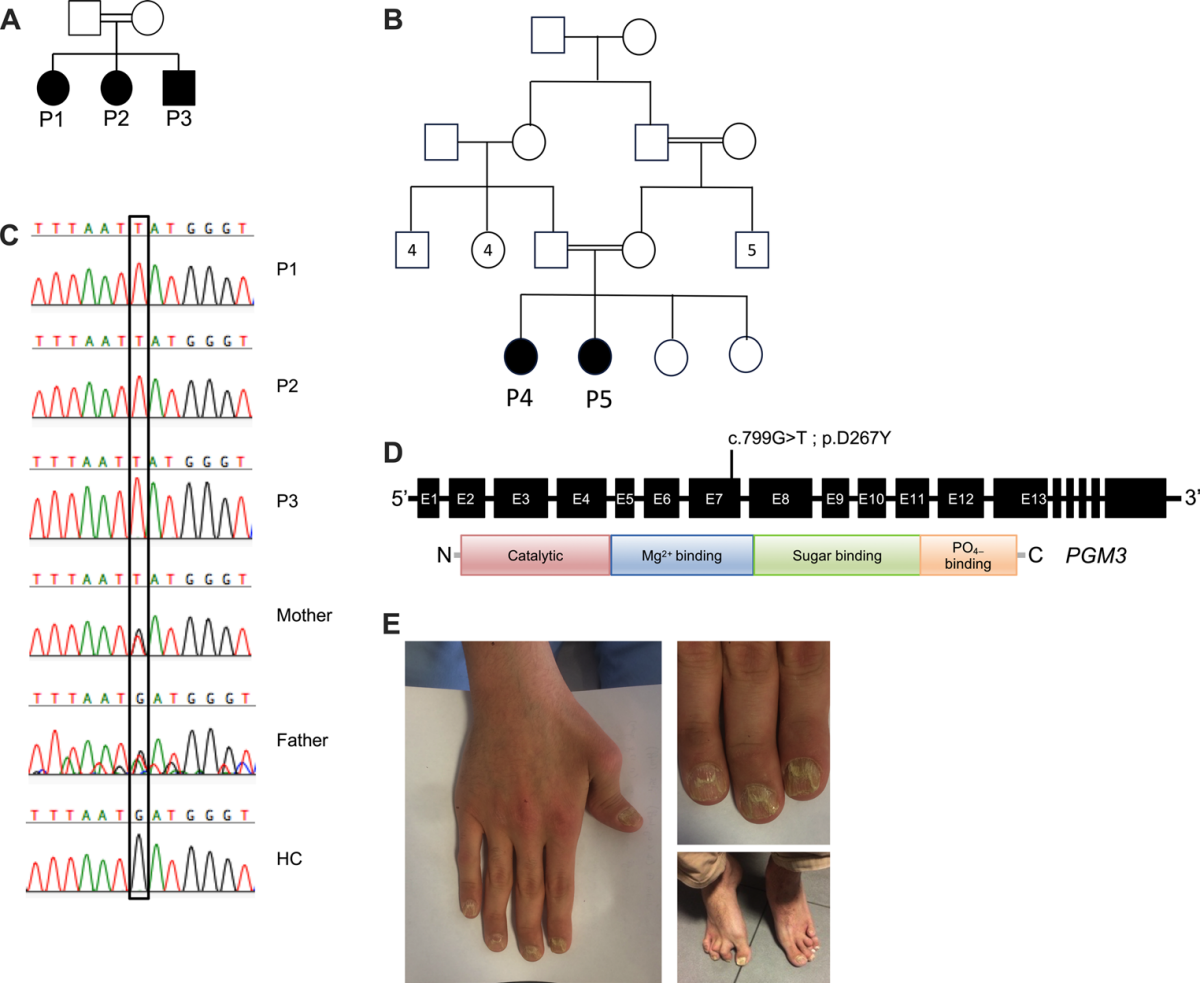
**A**-**B** Pedigrees of the two families. Black or white symbols represent individuals with or without clinical manifestations, respectively. C.Sequence analysis of a portion of exonic non-synonymous mutation in homozygous three patients, their heterozygous family members, and healthy control.D.Structure of the PGM3 gene: A schematic diagram of the PGM3 gene structure and mutation of the patients are noted according to its localization. E. Deviation of the metatarsophalangeal junction, degenerative changes, acetabular degeneration, pes planus, dislocation of the thumbs, Patient 3

The first family (Family A, P1, P2, P3) was a non-consanguineous family with three siblings presenting with early childhood onset combined immunodeficiency (CID). Their parents were both healthy with no histories of immunodeficiency, and only two infantile deaths were in the father’s family (Fig. 1A).

#### Patient 1 (P1)

**Fig. 2:**
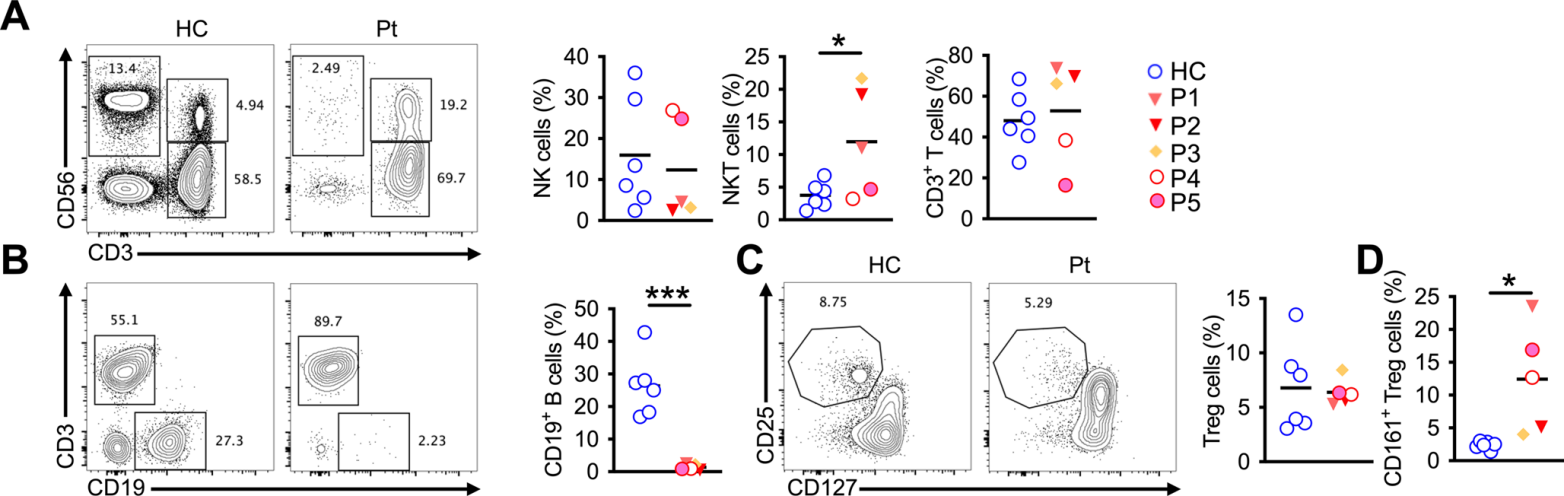
(**A**) FCA and cell frequencies of NK(CD56^+^ CD3^–^), NKT(CD56^+^ CD3^+^) and CD3 T-cells(CD56– CD3^+^CD8 T cells of P1, P2, P3, P4, P5 and six healthy controls. (**b**) FCA and cell frequencies of B cells(CD19+) of the respective groups. (**C**) FCA and cell frequencies of Treg cells(CD127^–^CD25^+^) of the respective groups. (**D**) Cell frequencies of CD161^+^ Treg cells of the respective groups. Each symbol represents one patient. Error bars indicate SEM. Statistical tests: Student t-test: *P < 0.05, ***P < 0.001. FCA: Flow cytometry analysis

is a 26-year-old female who was hospitalized for 5 days due to meconium aspiration in postnatal first hours. She had otorrhea since her newborn period. At 5 months of age, she presented with recurrent lower respiratory tract infections (LRTI), diaper dermatitis, moniliasis, seborrheic dermatitis, diarrhea, and eczematous rash at extensor sites from early infancy. Initial immunological work-up revealed low immunoglobulin A level, and intravenous immunoglobulin (IVIG) replacement treatment was started. Under IVIG replacement therapy, Recurrent skin abscesses and LRTI developed, but hospitalization was not required. Prior to referral to our clinic, the patient had been receiving G-CSF therapy for neutropenia for six years. At 21 years of age, she re-evaluated, and physical examination and immunological work-up revealed normal growth, alopecia areata, absent tonsils, eczematous rash on flexor sites, and neutropenia, lymphopenia, high IgE and IgA levels, decreased CD4/CD8 ratio, low CD4, B, and NK cells (Table 1; Fig. 2). The chest computed tomography (CT) scan evidenced bronchiectasis and millimetric pulmonary nodules. She had normal intelligence and good grades in school, and there was no evidence of skeletal dysplasia or dysmorphia (Table 1). Now, she is under IVIG replacement treatment without any infections or other clinical symptoms.

**Table 1:** Clinical and immunological findings

Age, years	P1	P2	P3		P4	P5
Age, years	**26**	**24**	**23**	**9**		**12**
Age of onset	**Newborn**	**7 months**	**Newborn**	**Newborn**		**1 year**
Clinical Findings						
Recurrent skin infections	**Skin abscesses**	**-**	**Furuncles**	**Skin abscesses**		
Recurrent sinopulmonary infections	**+**	**+**	**+** **(S.aureus)**	**+**		**+**
Otitis Media	**+**	**+**	**+**	**-**		**+**
Diarrhea	**+**	**+**	**-**	**-**		**-**
Diaper dermatitis	**+**	**-**	**-**	**-**		**-**
Seborrheic dermatitis	**+**	**-**	**-**	**-**		**-**
Fungal infections	**Moniliasis?**	**-**	**Onychomycosis**	**-**		**-**
Eczema	**+**	**-**	**+**	**-**		**-**
Food Allergy	**+**	**-**	**-**	**-**		**-**
Asthma	**-**	**+**	**-**	**+**		**+**
Allergic Rhinitis	**-**	**+**	**-**	**-**		**-**
Allergen Sensitization	**Negative**	**Cat-dog**,** HDM**,** tree-mix**,** pollens**	**Negative**	**Negative**		**HDM**
Viral infections	**-**	**Severe varicella inf**	**Rhinovirus and coronavirus pneumonia**	**-**		**-**
Skeletal dysplasia	**-**	**-**	**Deviation of metatarsophalangeal junction**,** acetabular degeneration**,** pes planus**,** dislocation of the thumbs**	**-**		**-**
Intellectual disability	**-**	**-**	**Mild**	**Mild**, **ADD**		**Moderate-low WISC-R score**
Neurodevelopmental delay	**-**	**-**	**-**	**-**		**Moderate**
Failure to thrive	**-**	**-**	**-**	**-**		**-**
Dysmorphic facial features	**-**	**-**	**Retrognathy**	**-**		**Mild coarse facial features**
Autoimmune manifestations	**-**	**-**	**JIA**	**-**		**-**
Other Clinical Findings	**-**	**-**	**Cataract**	**-**		**-**
Physical Examination						
Absent Tonsil	**+**	**+**	**+**	**-**		**-**
Hepatosplenomegaly	**-**	**-**	**Hepatomegaly**	**-**		**-**
Lymphadenopathy	**-**	**-**	**+**	**-**		**-**
Other Features	**Alopecia areata**	**-**	**Onychomycosis** **joint contractures**, **nail dystrophia**	**-**		**-**
Laboratory Data Peripheral Blood Count						
WBC (/mm^3^)	4700	**3900**,** ↓**	**2300**,** ↓**	**3500**,** ↓**		**3420**,** ↓**
Lymphocyte (/mm^3^)	**1100**,** ↓**	1900	**1000**,** ↓**	**1400**,** ↓**		1710
Neutrophil (/mm^3^)	**2000** **(under G-CSF)**	**600**,** ↓**	**400**,** ↓**	**1000**,** ↓**		**800**,** ↓**
Eosinophil (/mm^3^)	400	**700**,** ↑**	400	230		210
Lymphocyte Subset						
CD3^+^ n	1045, N	1824, N	**920**,** ↓**	**1022**,** ↓**		1426
CD4^+^n	**440**,** ↓**	760, N	**490**,** ↓**	798		634
CD8^+^n	495	969, N	400	420		759
CD4^+^/CD8^+^ratio	**0**,**88**	**0**,**78**	1,22	1,9		1,9
CD19^+^n	**11**,** ↓**	**19**,** ↓**	**10**,** ↓**	**182**,** ↓**		161
NK n	**55**,** ↓**	**57**,** ↓**	**70**,** ↓**	140		**92**,** ↓**
Naive T cells (CD45RA^+^) %	N/A	N/A	**17**	N/A		N/A
Memory T cells (CD45RO^+^) %	N/A	N/A	**83**	N/A		N/A
Serum Immunoglobulin						
IgG (mg/dl)	1028	859	1060	**375**,** ↓**		**563**,** ↓**
IgM (mg/dl)	175	**43**,** ↓**	**< 16**,** ↓**	**25.2**,** ↓**		**65**,** ↓**
IgA (mg/dl)	**564**,** ↑**	**529**,** ↑**	**247**,** ↑**	182.9		**435**,** ↑**
IgE (IU/L)	**372**,** ↑**	**322**,** ↑**	**1950**,** ↑**	39.2		84,5
Specific Antibody Response	N/A	N/A	N/A	Anti-Hbs: negative		Anti-Hbs: negative
Radiological Imaging						
Thorax CT scan	Bronchiectasis and nodules	Opacities atelectasis and nodules	Air cyst, bronchial wall thickening	N		N/A
Echocardiography	N/A	N/A	N/A	N		N/A
Abdominal Ultrasonography	N/A	N/A	Hepatomegaly	N		N/A
Spirometry	N	N	N/A	N/A		N/A

#### Patient 2 (P2)

had no history of infection until 7 months of age. Her first immunological evaluation was at 7 months of age following severe varicella infections with pneumonia, and IVIG replacement treatment was started. Her history was remarkable recurrent otitis media, LRTI, chronic diarrhea, asthma, and allergic rhinitis. The sensitivity of multiple aeroallergens was detected. Despite IVIG replacement therapy, infections could not be prevented. She had a normal physical examination except for absent tonsils. In re-evaluation of immunological parameters at 21 years of age, leukopenia, intermittent neutropenia (associated with infections), eosinophilia, increased IgE and IgA, decreased IgM, inverted CD4/CD8 ratio, B cell lymphopenia, and low NK cells were detected (Table 1; Fig. 2). Thorax CT scan revealed bilateral ground glass opacity areas, atelectasis, and millimetric calcified nodules. She had normal intelligence without skeletal anomalies, dysmorphia, or neurological symptoms (Table 1). Today, the patient is 24 years old and has fewer infections than in childhood under IVIG replacement treatments.

#### Patient 3 (P3)

is a 23-year-old male who presented with recurrent sinopulmonary infections and needed hospitalization from the first months of life. At the age of 3, he had lymphadenopathy. After this episode, the patient developed prolonged fever, arthralgia, and arthritis in the knee and was diagnosed with juvenile idiopathic arthritis treated with prednisolone and methotrexate. During the methotrexate treatment, leukopenia developed and resolved after discontinuation of treatment. His medical history was significant for mild atopic dermatitis, furuncle requiring drainage, cataract, and onychomycosis. The patient was treated with IVIG from the age of 3 until he turned 14. However, when he presented at 19 years of age, he was not receiving any treatment. On physical examination, dystrophic nails, mild deformities and limitation of movement of metacarpals (Fig. 1E), hepatomegaly, and absent tonsils were detected. Immunological studies showed neutropenia, CD4 lymphopenia, low B cells, and NK cells, with dysregulation of immunoglobulin levels (decreased IgM, increased IgE, and normal IgA and IgG). An inverted CD4/CD8 ratio was also detected with decreased naive T cells (Table 1; Fig. 2). Thorax CT scan revealed bronchial wall thickening. The patient had a mild mental disability and poor grades in school but no evidence of dysmorphia (Table 1). Today, he is mainly healthy under IVIG replacement treatment, besides recurrent bacterial upper airway infections, which have been treated with antibiotics.

#### The other two patients (Family B; P4 and P5)

were from a consanguineous family (Fig. 1B). Their parents and two sisters were healthy except for maternal epilepsy with no histories of immunodeficiency.

#### Patient 4 (P4)

is a 9-year-old female; at 13 days of age, she presented with pneumonia and required hospitalization. She had a remarkable medical history of recurrent fever, cough, and wheezing from early infancy and was diagnosed with asthma. The skin prick test was positive for house dust mites. Also, she suffered from a skin abscess that was treated with antibiotics without hospitalization. At 5 years of age, a routine blood test revealed leukopenia, neutropenia, T and B cells lymphopenia, decreased IgG and IgM, increased IgA and normal IgE, and adequate specific antibody responses (Table 1; Fig. 2). Evaluation by the WISC-R test only identified mild learning disabilities in the patients without neurological symptoms except for attention deficit disorder. No significant abnormalities were detected on the skeletal survey, abdominal ultrasound, thorax CT scan, and echocardiogram (Table 1).

#### Patient 5 (P5)

was evaluated at 12 years of age after her sister was diagnosed with PGM3 deficiency. The patient had recurrent upper respiratory tract infections during the first year of life. In her medical history, the patient had pneumonia twice, multiple wheezing episodes, and was diagnosed with asthma under control with low-dose inhaled corticosteroid (ICS). Skin prick test was positive for house dust mites. Immunological work-up revealed leukopenia, neutropenia, an inverted CD4/CD8 ratio, adequate specific antibody response, and dysregulation of immunoglobulins (Low IgG, normal IgM and IgE, and high IgA) (Table 1; Fig. 2). The patient had low WISC-R scores and moderate learning disabilities without neurological symptoms or dysmorphic features except for stuttering and mild coarse facial features (Table 1). Today, these two sisters are infection-free with IVIG treatment, and their asthma is controlled with ICS and montelukast treatments.

### Genetic and Functional Analysis of PGM3 Activity

Whole exome sequencing in the proband revealed homozygous exonic non-synonymous mutations in the PGM3gene PGM3 (NM_001199917.2): c.799G > T (p.Asp267Tyr). The variant was segregated within families 1 and 2 and was validated by Sanger sequencing of gDNA from peripheral leukocytes (Fig. 1C and D).

To our knowledge, the variant was not reported before in the literature; therefore, it is considered a novel variant and classified as a variant of unknown significance with some pathogenic evidence (PM5, PP3, PM2) according to the Varsome database [29].

**Fig. 3:**
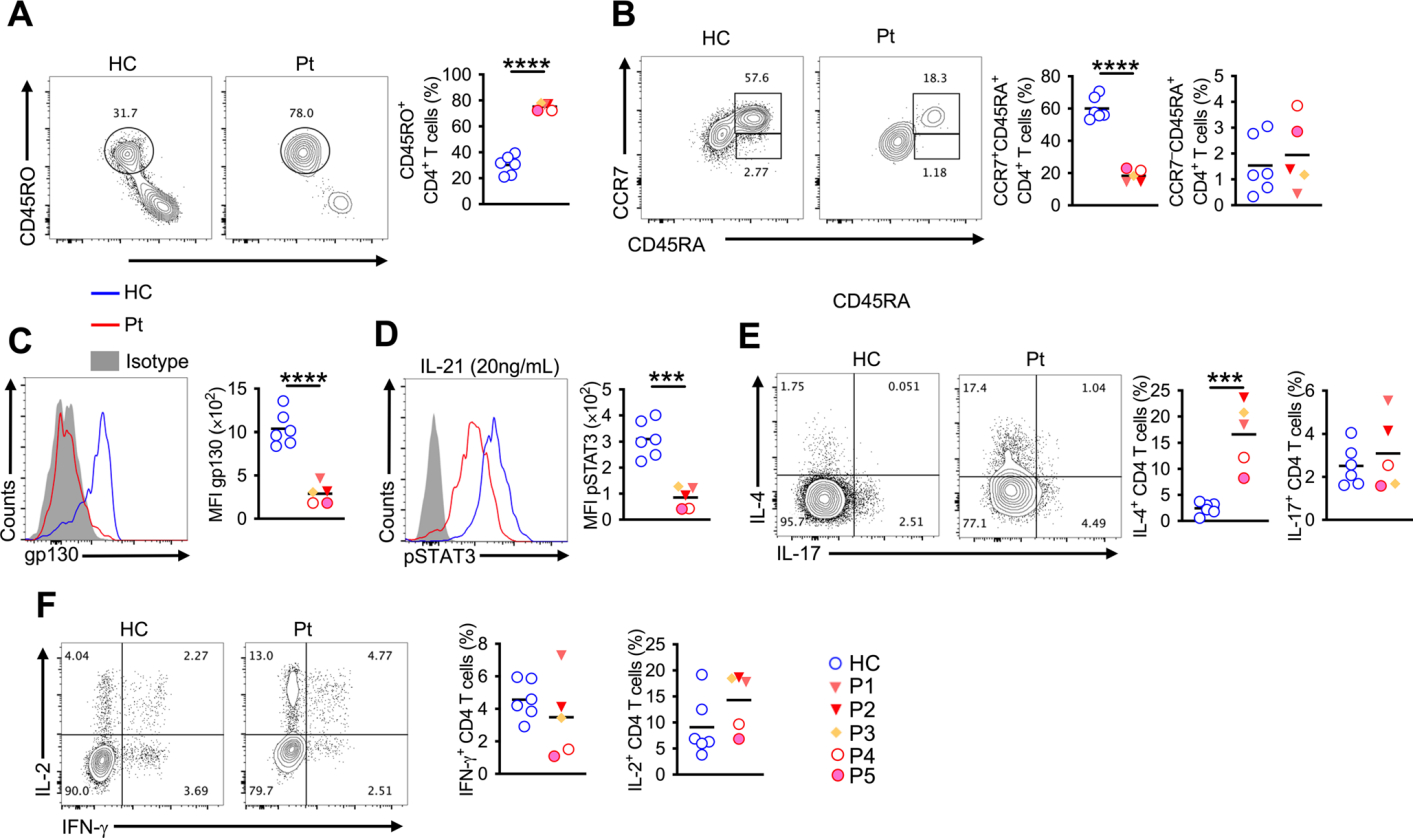
(**A**) FCA and cell frequencies of NK(CD56^+^ CD3^–^), NKT(CD56^+^ CD3^+^) and CD3 T-cells(CD56– CD3^+^CD8 T cells of P1, P2, P3, P4, P5 and six healthy controls. (**b**) FCA and cell frequencies of B cells(CD19+) of the respective groups. (**C**) FCA and cell frequencies of Treg cells(CD127^–^CD25^+^) of the respective groups. (**D**) Cell frequencies of CD161^+^ Treg cells of the respective groups. Each symbol represents one patient. Error bars indicate SEM. Statistical tests: Student t-test: *P < 0.05, ***P < 0.001. FCA: Flow cytometry analysis

**Fig. 4:**
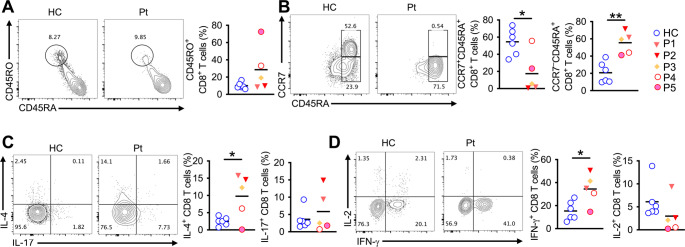
(**A**-**B**) Flow cytometry analysis and cell frequencies of naïve (CD45RA+ CCR7+), activated (CD45RO+) and TEMRA (CD45RA+ CCR7–) CD8 T cells of P1, P2, P3,P4, P5 and six healthy controls. (**C**-**D**) Flow cytometry analysis and cell frequencies of. IL-4, IFN-gamma, IL-17, and IL-2 secretion by CD8 T cells after 4 h of PBMCs culture with PMA (50 ng/mL; Sigma-Aldrich) and ionomycin (1 µL/mL; Sigma-Aldrich) stimulation of the respective groups. Each symbol represents one patient. Error bars indicate SEM. Statistical tests: Student t-test: *P < 0.05, **P < 0.01

Flow cytometry analysis revealed a statistically significant increase in NKT cells and a decrease in B cells in PGM3 patients compared with healthy controls; however, this difference was mainly driven by three patients from family A, while patients P4 and P5 showed values comparable to controls (Fig. 2A, B). Further analysis shows that patients present an increase in activated CD4^+^ T cells (CD45RO^+^), a decrease of naïve CD4^+^ and CD8^+^ T cells (CD45RA^+^ CCR7^+^), and an increase of TEMRA CD8^+^ T cells (CD45RA^+^CCR7^–^) (Figs. 3A-B and 4A-D-B). Functional analysis of patients revealed that the mutation affects the PGM3 function as shown by decreased surface expression of gp130, and p-STAT3 (Fig. 3C-D). These defects are associated with increased expression of IL-4 by CD4 and CD8 T cells and also an increased expression of IFN-γ by CD8 T cells. Specifically, IL-4 production by CD4⁺ T cells was increased in all PGM3 patients compared with healthy controls. In contrast, IL-4 production by CD8⁺ T cells was increased in patients P1–P4 but not in P5. Similarly, enhanced IFN-γ production was observed in CD8⁺ T cells from patients P1–P4, whereas patient P5 displayed levels comparable to healthy controls. No significant difference in IFN-γ production by CD4⁺ T cells was detected at the group level (Figs. 3E-F and 4C**- **D). Finally, analysis of regulatory T cells revealed no quantitative difference in regulatory-T cells frequencies; however, Tregs from patients with PGM3 deficiency exhibited increased pro-inflammatory potential, as evidenced by higher expression of CD161, a marker associated with pro-inflammatory cytokine-producing Treg subsets (Fig. 2D).

## Discussion

Herein, we describe five patients with an attenuated clinical presentation of PGM3 deficiency, characterized by a Hyper-IgE syndrome–like phenotype without skeletal dysplasia, dysmorphic features, or severe intellectual disability. One patient exhibited very mild skeletal dysplasia, and two patients had mild to moderate intellectual disability. Functional analyses demonstrated reduced gp130 surface expression and attenuated gp130-dependent STAT3 phosphorylation, consistent with partial impairment of downstream signaling. These functional alterations were accompanied by skewed T-cell cytokine profiles, including increased expression of IL-4 in CD4⁺ and CD8⁺ T cells and increased expression of IFN-γ in CD8⁺ T cells. Together, these findings extend previous observations by linking an attenuated clinical phenotype to partial functional impairment in PGM3 deficiency.

Most of the reported patients had the phenotype of classic glycosylation defects, including neurologic defects, dysmorphic features, and malformations. However, the phenotypical severity was variable, and some phenotypical features had not been seen in some patients. Also, some patients presented with phenotypical features of SCID and needed hematopoietic stem cell transplantation (HSCT) [6, 7, 12, 13]. Although elevated serum IgE levels represent a prominent and frequently reported feature of PGM3 deficiency [3, 5, 6, 9, 11, 16, 30], normal IgE levels have been described in a subset of patients [6, 7, 10, 16]. This variability suggests that IgE dysregulation is not an obligatory finding and may depend on the degree of residual PGM3 function, immune polarization, and disease stage. In line with previous studies indicating that PGM3 deficiency does not directly affect IgE glycosylation, normal IgE levels in some patients may reflect preserved regulation of Th2 responses or limited skewing toward Th2 differentiation [31]. Therefore, while high IgE levels are a helpful diagnostic clue, their absence does not exclude PGM3 deficiency. Moreover, contrary to the previous hypothesis that normal IgE levels might be attributed to early age, as suggested by the normal IgE levels in some newborn STAT3-deficient patients, normal IgE levels were found in two of our patients (9 and 12 years old). In addition, the IgE levels of our two adult patients were not as high as expected.

In contrast to classical congenital disorders of glycosylation, serum transferrin and apolipoprotein profiles remain normal in PGM3 deficiency, limiting their diagnostic utility and further highlighting the immunological rather than systemic glycosylation-driven nature of the disease [8].Therefore, there are still no established phenotypical or biological markers for PGM3.

As a result of hypomorphic PGM3 mutations causing abnormal glycosylation of many glycoproteins, the patients had variable immunological phenotypes, including neutropenia [3, 5–7, 12], lymphopenia [3, 5–16], eosinophilia [5, 10, 11, 16], low counts of T cells and B cells [3, 5–16], decreased NK cells [3, 10, 12, 16] and variable immunoglobulin levels [3, 5–16]. In addition to variable immunological parameters, dysmorphic features [5–8, 13, 14], skeletal dysplasia [3, 5–8, 12, 13, 16], and neurodevelopmental impairments [3, 5–7, 9, 12, 13, 16] seen in patients with PGM3 deficiency. A comprehensive overview of the clinical and immunological characteristics of previously reported patients is provided in Table S2.

Depending on their immunological disorders, most patients suffer from mild to severe infections due to bacterial, viral, or fungal causes, as in other combined immunodeficiency [9]. Also, our patients presented with bacterial and viral infections, and one had chronic mucocutaneous candidiasis. Although atopic phenotypic features have been seen in the patients, sensitization of the allergens might not be shown in some patients, similar to the present study.

While initial reports suggested that milder osseous conditions like scoliosis and hyper-extensibility were more prevalent than severe skeletal dysplasia [3, 5], following reports show that skeletal dysplasia was very variable [6–16]. Similar to our patients, some patients had no skeletal dysplasia [8]. Dysmorphic features, especially the downturned corner of the mouth, midface hypoplasia, anteverted nostrils, and micrognathia, have also been reported [5–8, 13, 14]. Previous studies reported that these typical dysmorphic traits could be associated with disease severity [13]. Indeed, the absence of dysmorphic features and milder clinical manifestations in our patients supports this hypothesis.

Glycosylation defects, including patients with PGM3 deficiency, often cause neurological abnormalities and neurodevelopmental delay [32]. Neurological findings and neurodevelopmental delay were observed in all patients with the SCID phenotype, but this was not true for patients with the HIES phenotype. However, there does not appear to be a relationship between the degree of immunological impairment and the severity of neurological findings [3, 5–16]. In this study, patients P1 and P2 experienced a history of more severe infections compared with P3 and P5; however, their neurodevelopmental status was preserved, and no neurological abnormalities were identified.

In previous reports, when considering the critical role of glycosylation in developing hematological and immunological cells, they reported that disease severity could affect these abnormalities. All previously described severely affected patients with PGM3 deficiency were neutropenic except one patient [13–15]. However, all our patients presented with neutropenia despite their mild phenotype.

In previous studies, some investigators have tried to establish a correlation between genotype and phenotype or the enzyme level and phenotype [8, 10, 15, 16, 31]. Winslow et al. [15] reported that, similar to the case in the report of Lundin et al., the patient with p.Ile350Thr without neurological and skeletal abnormalities [10]. Moreover, they suggested that although the p.Arg520Ter nonsense variant has unclear phenotypic features, the patients with mutations in the phosphate-binding domain of PGM3 had severe T-cell lymphopenia but lacked abnormalities of the nervous or skeletal systems [10, 16]. Ben-Khemis et al. reported milder clinical phenotypes associated with certain missense mutations; however, this association was not consistent across all missense variants [16]. In a previous report, ALG12 polymorphism seemed to affect the different clinical phenotypes observed in two patients, similar to previous cases with other CGD or IEI [8, 33]. However, there is a need for further studies to explain the correlation between both of these/other genes involved in N-glycosylation, which might cause differences in the severity of PGM3 deficiency.

Ben-Ali et al. demonstrated that patients with PGM3 deficiency exhibit compromised STAT3 signaling downstream of the extensively glycosylated protein gp130, the common signal-transducing receptor subunit for IL-6–type cytokines, providing a mechanistic basis for overlapping clinical features observed in PGM3 deficiency and AD-HIES [19]. Similar findings were reported in a gp130-deficient patient presenting with AD-HIES–like features, who showed impaired STAT3 phosphorylation in response to IL-6 family cytokines such as IL-27 but not to gp130-independent cytokines including IL-10 or IL-21 [34]. In our cohort, we observed diminished STAT3 phosphorylation following IL-21 stimulation (Fig. 4B), indicating partial impairment of STAT3 signaling. Given that IL-21 primarily signals via the IL-21 receptor and the common γ-chain, this finding should not be interpreted as direct evidence of gp130 dysfunction but rather as supportive of broader alterations in STAT3 activation in PGM3 deficiency. Gp130 was weakly expressed on PGM3-deficient cells due to impaired glycosylation [16, 19]. Our study demonstrated lower gp130 expression in peripheral T cells(Fig. 4B). The previous report detected that patients with PGM3 deficiency with different mutations and different phenotypes had different expressions of gp130. Although, patients with severe phenotypes were especially attributed to the more profound impairment of glycosylation [19], gp130 expressions of our patients were significantly impaired.

Previous ex vivo studies have reported preserved IL-17–producing T-cell function in PGM3 deficiency [16]; which is supported by our intracellular cytokine staining data showing no major defect in IL-17–producing cells. However, impaired IL-17 responses have been described in association with specific PGM3 variants, such as Glu340del, indicating variant-dependent functional heterogeneity [19]. IL-2 is known to play a complex regulatory role in T-cell lineage commitment, including inhibitory effects on Th17 and T follicular helper differentiation, while supporting other T-helper cell programs [35]. Nevertheless, in the present study, IL-2 expression was not uniformly increased across patients and was not quantified in culture supernatants; therefore, a direct contribution of IL-2 to the preserved Th17 phenotype cannot be conclusively established.

In parallel, the altered balance of CD4⁺ T-cell subset polarization observed in another cohort implies that normal PGM3 function is required not only to restrain overactive Th1/Th2 responses but also to support adequate regulatory and Th17 compartment integrity, thereby maintaining immune homeostasis. Hyperactive Th2 responses together with functionally altered Tregs are likely to contribute to elevated serum IgE levels, eosinophilia, and eczema, while impaired Th17 differentiation may further explain the susceptibility to bacterial and fungal infections [31]. Together, these findings indicate that PGM3 deficiency affects both effector and regulatory arms of CD4⁺ T-cell immunity, resulting in an imbalanced inflammatory milieu. Moreover, in our patients, B-cell lymphopenia was a prominent and consistent finding. B-cell development and survival are highly dependent on tightly regulated surface receptor expression and turnover, processes that may be particularly sensitive to disruptions in UDP-GlcNAc availability and N-linked glycosylation [36].

Additionally, Ben-Khemis et al. reported no quantitative difference in FOXP3⁺ CD4⁺ T-cell frequencies between patients with PGM3 deficiency and healthy controls [16]. Consistent with these observations, overall Treg frequencies were preserved in our cohort. However, we identified a distinct subset of Tregs displaying a pro-inflammatory phenotype, characterized by increased expression of CD161, a marker previously associated with cytokine-producing, functionally altered Treg populations. While pro-inflammatory Treg subsets have been described in other inflammatory and immune-mediated conditions, their presence and potential contribution in the context of PGM3 deficiency have not been previously emphasized. This finding suggests qualitative alterations in regulatory T-cell function rather than a numerical defect.

The presence of functionally skewed Tregs in our cohort further supports the concept that PGM3 deficiency impacts immune regulation predominantly at a qualitative level within the T-cell compartment. In this context, the heterogeneous clinical manifestations observed in patients with PGM3 deficiency -including autoimmunity, malignancy, and prominent atopic disease- may reflect a broader immune dysregulation phenotype that extends beyond classical HIES features and CID alone. Although our data do not provide direct functional proof of defective immune regulation, the identification of a pro-inflammatory Treg profile suggests impaired regulatory control and may represent one contributing factor to the diverse clinical outcomes observed in PGM3 deficiency. Additionally, our findings reveal a nuanced pattern of T-cell dysregulation in patients with PGM3 deficiency. While IL-4 production by CD4⁺ T cells was consistently increased across all patients, cytokine alterations in CD8⁺ T cells showed greater inter-individual variability, with increased IL-4 and IFN-γ production observed in most patients but not uniformly present in all cases. This heterogeneity suggests that immune dysregulation associated with PGM3 variants may be influenced by additional genetic, epigenetic, or environmental modifiers. Such variability is consistent with the broad clinical and immunological spectrum previously described in PGM3 deficiency and may contribute to the milder phenotype observed in our cohort.

In conclusion, the mechanisms underlying atopic manifestations in PGM3 deficiency remain incompletely understood. Our findings are consistent with previous reports showing that defective glycosylation leads to reduced gp130 surface expression and attenuated gp130-dependent STAT3 phosphorylation. In particular, we observed impaired IL-21–induced STAT3 activation, supporting partial disruption of gp130-dependent signaling. Importantly, the functional cellular features observed in our patients partially overlap with those described in STAT3 loss-of-function and gp130 deficiency, while remaining clinically distinct. Together, our data extend existing knowledge by linking an attenuated clinical phenotype to partial functional impairment of gp130–STAT3 signaling in PGM3 deficiency. Further studies in larger cohorts are required to better define genotype–phenotype relationships and modifiers of disease severity.

## Supplementary Information

Below is the link to the electronic supplementary material.

Supplementary Material 1 (DOCX 474 KB)

Supplementary Material 2 (DOCX 474 KB)

Supplementary Material 3 (XLSX 4.70 MB)

Supplementary Material 4 (XLSX 4.70 MB)

## Data Availability

The datasets generated and/or analyzed during the current study are available from the corresponding author on reasonable request.
