# Supplementary material for: A Novel Phosphoglucomutase-3 Gene Variant Causing Milder Phenotype in Two Families
Source: J Clin Immunol. 2026 Mar 29;46(1):49. doi: 10.1007/s10875-026-02012-2 (PMC13156099; doi:10.1007/s10875-026-02012-2)
Supplement: Supplementary file 1 — Supplementary Material 1 (DOCX 474 KB) [file 10875_2026_2012_MOESM1_ESM.docx]

**A Novel Phosphoglucomutase-3 Gene Variant Causing Milder Phenotype in Two Families**

**Table S1: Summary of candidate variants after multi-step filtering and ACMG-based evaluation.**

| **Gene** | **Chromosome** | **Coordinate** | **Genotype** | **Functional Annotation** | **Exonic Function** | **cDNA Change** | **dbSNP ID** | **OMIM ID** | **OMIM Phenotype** |
| --- | --- | --- | --- | --- | --- | --- | --- | --- | --- |
| PGM3 | Chr6 | 83891527 | homozygous | exonic | Nonsynonymous SNV | NM_001199917.1:c.799G>T:p.D267Y | **.** | 172100 | Immunodeficiency 23;AR;615816 |
| TYK2 | Chr19 | 10478837 | heterozygous | exonic | Nonsynonymous SNV | NM_003331.4:c.359C>T:p.P120L | rs758463317 | 176941 | Immunodeficiency 35;AR;611521 |

**Figure S1: Gating strategy for Treg cells in human peripheral blood mononuclear cells (PBMCs).**

**Figure S2: Gating strategy for p-STAT3 analysis.**

**Table S2. Summary of Previously Reported Cases of PGM3 Deficiency in the Literature and Their Clinical Spectrum.**

| Patient ID  and references | Genetic | Age (y), gender | Clinical manifestations | Immunological parameters |
| --- | --- | --- | --- | --- |
| Zang et al^3^ I.1 | c.1585G>C,  p. E529Q | 35, M | *Asthma, allergic rhinitis, atopic dermatitis  *Vasculitis, EMM  *Rec RTI, recurrent staphylococcal skin infections, bronchiectasis  *Neurodevelopmental impairments  *Scoliosis, degenerative disc disease, dilated aortic root  *MPGN on hemodialysis | * Lymphopenia  * CD3+ T cells low  * Low CD27^+^ memory B cells  *Increased IgE and IgG |
| Zang et al^3^ I.2 | c.1585G>C,  p. E529Q | 32, F | *Food allergy, allergic rhinitis,  drug allergy, atopic dermatitis,  *Vasculitis, EMM,  *EBV viremia, rec RTI, bronchiectasis s/p bilateral  lung transplantation  *Neurodevelopmental impairments  *Esophageal stricture | * Lymphopenia  * CD3^+^ T cells low  * Low CD27^+^ memory B cells  * Increased IgE, IgG and IgA |
| Zang et al^3^ I.3 | c.1585G>C,  p. E529Q | 30, M | *Asthma, food allergy, atopic dermatitis,  *Vasculitis, EMM,  *EBV viremia, rec RTI, molluscum,  recurrent staphylococcal skin infections  *Neurodevelopmental impairments  *Scoliosis, esophageal diverticulitis  *Hodgkin lymphoma (EBV+) | * Lymphopenia  * CD3^+^ T cells low  * Low CD27^+^ memory B cells  * Increased IgE and IgA |
| Zang et al^3^ I.4 | c.1585G>C,  p. E529Q | 30, M | *Food allergy, drug allergy, Atopic dermatitis,  *Vasculitis, EMM,  *EBV viremia, otitis, recurrent staphylococcal skin infections, bronchiectasis  *Neurodevelopmental impairments  * Scoliosis  *MPGN  * Hodgkin lymphoma (EBV+) | * Lymphopenia  * CD3^+^ T cells low  * Low CD27^+^ memory B cells  * Increased IgE and IgA |
| Zang et al^3^ I.5 | c.1585G>C,  p. E529Q | 27, F | *Asthma, allergic rhinitis, Atopic dermatitis, eczema herpeticum,  *EMM  *HSV esophagitis, rec RTI, bronchiectasis  *Neurodevelopmental impairments  * Esophageal stricture | * Lymphopenia  * Neutropenia (Antineutrophil antibody positive)  * Increased IgE |
| Zang et al^3^ II.1 | c.975T>G,  p.D325E | 12, M | *Food allergy, allergic rhinitis,  drug allergy, atopic dermatitis,  *Recurrent staphylococcal skin infections, fungal pneumonia, rec RTI  *Neurodevelopmental impairments  *Microcephaly, scoliosis  *Hepatosplenomegaly  *Unilateral agenesis | * Lymphopenia  *Hemolytic anemia  * CD3^+^ T cells low  *Low CD27^+^ memory B cells  * Increased IgE, IgG and IgA |
| Zang et al^3^ II.2 | c.975T>G,  p.D325E | 10, M | *Allergic rhinitis, Atopic dermatitis,  *Flat warts, recurrent staphylococcal skin infections, otitis  *Neurodevelopmental impairments | * Lymphopenia  * Low CD27^+^ memory B cells  * Increased IgE, IgG and IgA |
| Zang et al^3^ II.3 | c.975T>G,  p.D325E | 1, M | *Food allergy, FPIES, atopic dermatitis,  *RSV, recurrent staphylococcal skin infections, otitis,  *Neurodevelopmental impairments | * Lymphopenia  * Neutropenia  * CD3^+^ T cells low  *Low CD27^+^ memory B cells  * Increased IgE and IgA |
| Sassi et al^5^ and Ben Khemis et al^16^ A.V.12 | c.1018_1020del;  p.E340del | 7, M | *Rec RTI, abscesses, candidiasis, *S. aureus* infections  *Neurodevelopmental impairments  *Scoliosis | *Eosinophilia  *Neutropenia  * CD4^+^ T cells and B cells low  *Increased IgE |
| Sassi et al^5^ and Ben Khemis et al^16^ A.V.13 | c.1018_1020del;  p.E340del | 14 mo, F | *Rec RTI, abscesses, candidiasis, RSV, *S. aureus* infections | * Eosinophilia  * CD3^+^ T cells and CD4^+^ T cells low  *Increased IgE |
| Sassi et al^5^ and Ben Khemis et al^16^ A.V.14 | c.1018_1020del;  p.E340del | 13 mo, F | *Rec RTI, abscesses, candidiasis, RSV, *S. aureus* infections | * Lymphopenia  * Neutropenia  * Eosinophilia  * CD3^+^ T cells, CD4^+^ T cells and B cells low  *Increased IgE, IgG, IgM and IgA |
| Sassi et al^5^ A.V.18 | c.1018_1020del;  p.E340del | 6, M | *Rec RTI, abscesses, candidiasis, *S. aureus* infections  *Hyperextensibility  *Neurodevelopmental impairments | *Eosinophilia  * CD3^+^ T cells, CD4^+^ T cells and B cells low  *Increased IgE |
| Sassi et al^5^ B.V.6 | c.248T>C;  p.L83S | 34, M | *Rec RTI, abscesses, candidiasis, *S. aureus* infections  * Characteristic facies, Hyperextensibility  *Neurodevelopmental impairments | *Eosinophilia  * CD3^+^ T cells and CD4^+^ T cells low  *Increased IgE and IgA |
| Sassi et al^5^ BV.7 | c.248T>C;  p.L83S | 32, M | *Rec RTI, abscesses, candidiasis, *S. aureus* infections  *Characteristic facies, Hyperextensibility  *Neurodevelopmental impairments | *Eosinophilia  * CD3^+^ T cells and CD4^+^ T cells low  *Increased IgE, IgG, IgM and IgA |
| Sassi et al^5^ C.IV.7 | c.1504G>T;  p.D502Y | 11.5, M | *Rec RTI, severe VZV, *S. aureus* infections  *Neurodevelopmental impairments | *Lymphopenia  *Eosinophilia  *B cells low  *Increased IgE and IgA, low IgG |
| Sassi et al^5^ D.IV.2 | c.248T>C;  p.L83S | 21, M | *Rec RTI, Abscesses, *S. aureus* infections  * Characteristic facies  *Neurodevelopmental impairments | *Eosinophilia  *Increased IgE |
| Sassi et al^5^ D.IV.5 | c.248T>C;  p.L83S | 11, M | *Rec RTI  *Characteristic facies  *Neurodevelopmental impairments | *Eosinophilia  *Increased IgE |
| Stray-Pederson et al^6^ P1 | c.737A>G;  p.Asn246Ser | 1.5, F | *Eczema  *Rec RTI, skin infections  *Skeletal dysplasia, hydrocephalus  *Dysmorphic facial features, developmental delay | *T- B- NK+ SCID  *Neutropenia  *Low IgM and IgA, normal IgG and IgE |
| Stray-Pederson et al^6^ P2 | Compound  heterozygous,  c.715G>C and chr6.hg19:g.(83,  013,454_83,145,962)(84,389,166_  84,395,825)del  p.Asp239His | 6.5, M | *Eczema  *Rec RTI, skin infections | *T- B- NK+ SCID  *Neutropenia  *Low IgM, normal IgG and IgA, high IgE |
| Stray-Pederson et al^6^ P3 | Compound  heterozygous,  c.737dupA;c.1352A>G,  p.Asn246Lysfs*7;p.Gln451Arg | 7 mo, M | *Eczema  *Rec RTI, skin infections  *Skeletal dysplasia, hydrocephalus  *Dysmorphic facial features, developmental delay | *T- B- NK+ SCID  *Neutropenia  *Normal IgM, IgA, and IgE, low IgG from 3 months |
| Bernth-Jensen et al^7^ | c.737A>G;  p.Asn246Ser | 0, M | *Sepsis with *S. epidermidis* and coagulase-  negative species  *Dysmorphic facial features  *Horseshoe kidney, atrial septal defect,  intestinal malrotation, and bilateral exaggerated trochanter minor | * Lymphopenia  *T- B- NK+ SCID  * Neutropenia  * Low IgG,IgA and IgE  * Normal IgM |
| García-García et al^8^  P1 | Compound  heterozygous,  c.1391C>T/c.1432C>T  p.T464I; p.Q478X | 23, F | *Eczema  *Otitis media, invasive candidiasis, HPV  *Scoliosis | *Lymphopenia  *CD4^+^ T cells low  *Eosinophilia  *Increased IgE and IgA |
| García-García et al^8^  P2 | Compound  heterozygous,  c.1391C>T/c.1432C>T  p.T464I; p.Q478X | 11, F | *Eczema, food and drug allergy, asthma  *Otitis media, non-invasive candidiasis, recurrent staphylococcal skin infections, HPV  *Dysmorphic facial features | * Lymphopenia  * CD4^+^ T cells low  *Eosinophilia  *Increased IgE and IgA |
| Ittiwut et al^9^ | Compound  heterozygous,  c.1003A>G (p.Thr335Ala)  c.1443delC (p.Asn482Metfs*4) | 9, M | *Severe atopic dermatitis, multiple food allergies  *Chronic diarrhea  *Rec RTI, salmonella and candida septicemia, a severe varicella infection | * Lymphopenia  * Neutropenia  * Eosinophilia  * CD3^+^ T cells, CD4^+^ T, CD8^+^ T cells low and very low B cells  * Increased IgE and IgG |
| Lundin et al^10^ H.D. | c.965 T>C  p. I322T | 12, F | *Persistent eczema  *Recurrent skin abscesses, otitis media, bronchitis, severe varicella infection  *Arthritis | * Neutropenia  * Eosinophilia |
| Lundin et al^10^ N.D. | c.965 T>C  p. I322T | 10, M | *Eczema  *Gastroenteritis, gluteal abscess (*S. aureus* and *C. albicans)* and Rec RTI | * Neutropenia  * Eosinophilia |
| Lundin et al^10^ E.D. | c.965 T>C  p. I322T | 16, F | *Eczema  *Thoracic skin abscess (*S. Aureus*), severe perianal dermatitis, Rec RTI and severe varicella infection | N/A |
| Lundin et al^10^ A.D. | c.965 T>C  p. I322T | 43, F | *Eczema (mild)  *Severe varicella infection, Rec RTI | * Neutropenia  * Eosinophilia  * CD3^+^ T cells,and B cells low  * Increased IgA |
| Lundin et al^11^ | c.871+3A > G | 3, F | *Eczema, severe food allergy  *Autoimmunity: TSH receptor and TPO autoantibodies positive  *Multiple abscesses, Rec RTI, rec S. aureus and candida infections, severe CMV infection  * Failure to thrive | *Lymphopenia  *Eosinophilia  * CD3^+^ T cells and CD4^+^ T cells low  *Increased IgE, IgG, IgM and IgA |
| Pacheco-Cuellar et al^12^ Case 1 | c.1135T>C  p.Phe379Leu | 7 d, M | *Respiratory insufficiency and pulmonary hypertension  *Skeletal dysplasia  *Dysmorphic facial features | *T- B- NK+ SCID  * Neutropenia  * Thrombocytopenia |
| Pacheco-Cuellar et al^12^ Case 2 | c.1135T>C  p.Phe379Leu | 5 d, F | * Intestinal obstruction, persistent pulmonary hypertension  * Skeletal dysplasia  * Dysmorphic facial features  * Splenomegaly  * Horseshoe kidney | *T- B- NK+ SCID  * Neutropenia  * Thrombocytopenia |
| Fusaro et al^13^ P1 | c.146G>C  p.Arg49Thr | 16 mo, M | *Cutaneous S.aureus infection, cervical adeniti, sepsis  *Multiple skeletal abnormalities  * Neurological impairment | *Severe T cell lymphopenia  *Impaired T cell proliferation |
| Fusaro et al^13^ F1 | c.1268 T>C  p.Met423Thr | F | *Facial dysmorphia  *Severe cerebral abnormalities  *Multiple skeletal abnormalities  *Horseshoe kidneys | N/A |
| Fusaro et al^13^ P2 | c.1268 T>C  p.Met423Thr | 3 mo, F | *Dysmorphic facial features  *Multiple skeletal abnormalities  **S. epidermidis* infection | *Agranulocytosis and severe T cell lymphopenia  *Low IgE |
| Fallahi et al^14^ Patient 1 | c.845T>C | 32 mo, M | *Eczema, food allergy  *Dysmorphic facial features  *Bullous pemphigoid  *Oral candidiasis, fungal skin infection, recurrent otitis media  *Delayed umbilical cord separation | * Lymphopenia  * Neutropenia  * CD4^+^ T cells and B cells low  * Increased IgE and IgM |
| Fallahi et al^14^ Patient 2 | c.845T>C | 1, F | *Eczema, food allergy  *Dysmorphic facial features  *Bullous pemphigoid  *Delayed umbilical cord separation  *Oral candidiasis, Rec RTI | * Lymphopenia  * CD3^+^ T cells and CD8^+^ cells low  * Increased IgE and IgA |
| Winslow et al^15^ | Compound  heterozygous,  c.1049 T>6,  p.Ile350Thr;  c.1558C>T,  p.Arg520Ter | 13 mo, F | *Oral and diaper candidiasis | *Abnormal T-cell receptor excision circle assay  * Leukopenia  * Lymphopenia  * Neutropenia  * CD3^+^ T cells, CD4^+^ T cells, CD8^+^ cells and B cells low  * Low IgM and IgA |
| Ben Khemis et al^16^  P4 | c.1018_1020del  p.Glu340del | 5, F | *Eczema  *Rec RTI, Staphylococcus infections, cutaneous abcesses, candidiasis  *Scoliosis  * Neurological impairment | * Lymphopenia  * CD3^+^ T cells, CD4^+^ T cells, CD8^+^ cells and B cells low  * Increased IgE |
| Ben Khemis et al^16^  P5 | c.1018_1020del  p.Glu340del | 16, M | *Eczema  *Rec RTI, cutaneous abcesses  *Scoliosis  * Neurological impairment | * Increased IgE |
| Ben Khemis et al^16^  P6 | c.1018_1020del  p.Glu340del | 7, F | *Rec RTI, cutaneous abcesses  * Neurological impairment | * Increased IgE |
| Ben Khemis et al^16^  P7 | c.1018_1020del  p.Glu340del | 15, M | *Eczema  *Rec RTI  *Scoliosis  * Neurological impairment | N/A |
| Ben Khemis et al^16^  P8 | c.1018_1020del  p.Glu340del | 11, F | *Eczema  *Rec RTI, cutaneous abcesses  * Neurological impairment | * Lymphopenia  * CD3^+^ T, CD4^+^ T and B cells low  * Increased IgE |
| Ben Khemis et al^16^  P9 | c.1018_1020del  p.Glu340del | 17, M | *Eczema  *Rec RTI, HPV  *Scoliosis  * Neurological impairment | * Increased IgE |
| Ben Khemis et al^16^  P10 | c.1018_1020del  p.Glu340del | 1, F | * Cutaneous abcesses *(Pseudomonas aeruginosa)*  * Neurological impairment | *Eosinophilia  * CD4^+^ T cells and B cells low |
| Ben Khemis et al^16^  P11 | c.1018_1020del  p.Glu340del | 4, F | *Eczema  *Staphylococcal scalded skin syndrome, cutaneous abcesses  * Neurological impairment | * Lymphopenia  * CD3^+^ T, CD4^+^ T cells, CD8^+^ T cells and B cells low  * Increased IgE |
| Ben Khemis et al^16^  P12 | c.1018_1020del  p.Glu340del | 3, M | *Eczema  *Rec RTI, cutaneous abcesses *(Pseudomonas aeruginosa, candidiasis* | * Lymphopenia  * CD3^+^ T, CD4^+^ T cells, CD8^+^ T cells and B cells low  *Eosinophilia |
| Wang et al^18^ I.1^a^ | Compound  heterozygous,  c.1585G>C,  p.Glu529Gln;  c.1438_1442del,  p.Leu480SerfsTer10 | 47, M | *Atopic dermatitis  *Rec RTI, bronchiectasis  *Neurological impairment  *Scoliosis,  *MPGN, cutaneous leukocytoclastic vasculitis  *Nonischemic cardiomyopathy | * Increased IgE |
| Wang et al^18^ I.2 | Compound  heterozygous,  c.1585G>C,  p.Glu529Gln;  c.1438_1442del,  p.Leu480SerfsTer10 | 41, F | *Atopic dermatitis, food allergy  *Rec RTI, bronchiectasis, chronic norovirus infection  *Neurological impairment  *Scoliosis,  *Cutaneous leukocytoclastic vasculitis  *EBV-positive DLBCL | *Neutropenia  *Increased IgE |
| Wang et al^18^ I.3 | Compound  heterozygous,  c.1585G>C,  p.Glu529Gln;  c.1438_1442del,  p.Leu480SerfsTer10 | 37, M | *Atopic dermatitis, food allergy, eosinophilic esophagitis  *Rec RTI, bronchiectasis, molloscum contagiosum,  HSV-1 infection  *Neurological impairment  *Scoliosis  *Cutaneous leukocytoclastic vasculitis  *EBV-positive Hodgkin lymphoma, DLBCL | *Neutropenia  *Increased IgE |
| Wang et al^18^ I.4 | Compound  heterozygous,  c.1585G>C,  p.Glu529Gln;  c.1438_1442del,  p.Leu480SerfsTer10 | 42, M | *Atopic dermatitis, food allergy, eosinophilic esophagitis  *Rec RTI, bronchiectasis, molloscum contagiosum,  HSV-1 infection  *Neurological impairment  *Scoliosis  *MPGN, Cutaneous leukocytoclastic vasculitis  *EBV-positive Hodgkin lymphoma; right eye SCC | * Increased IgE |
| Wang et al^18^ II.1 | c.975T>G,  p.Asp325Glu | 24, M | *Atopic dermatitis, food allergy, allergic Bronchopulmonary aspergillosis  *Rec RTI, bronchiectasis  *Neurological impairment  *Scoliosis  ,*Psoriasis | *Neutropenia (with positive anti-neutrophil antibody)  * Increased IgE |
| Wang et al^18^ II.2 | c.975T>G,  p.Asp325Glu | 18, M | *Atopic dermatitis, food allergy  *Rec RTI, verruca plana  *Neurological impairment  *Scoliosis  *Coombs-negative hemolytic anemia | *Neutropenia  * Increased IgE |
| Wang et al^18^ II.3 | c.975T>G,  p.Asp325Glu | 13, M | *Atopic dermatitis, food allergy, allergic fungal otomastoiditis  *Rec RTI  *Neurological impairment | *Neutropenia  *Increased IgE |
| Wang et al^18^ III.1 | c.982G>A,  p.Ala328Thr | 11, F | * Atopic dermatitis, food allergy  *Rec RTI | *Neutropenia  *Increased IgE |
| Wang et al^18^ IV.1 | Compound  Heterozygous  c.1592C>T  p.Ala531Val;  c.620C>G,  p.Ser207Ter | 6, M | * Atopic dermatitis, food allergy  * Molloscum contagiosum | * Increased IgE |
| Wang et al^18^ IV.2 | Compound  Heterozygous  c.1592C>T  p.Ala531Val;  c.620C>G,  p.Ser207Ter | 8, F | * Atopic dermatitis, food allergy  *Rec RTI  * Molloscum contagiosum | *Neutropenia  *Increased IgE |
